# *Bmp2*, *Bmp4* and *Bmp7* Are Co-Required in the Mouse AER for Normal Digit Patterning but Not Limb Outgrowth

**DOI:** 10.1371/journal.pone.0037826

**Published:** 2012-05-25

**Authors:** Kyung-Suk Choi, Chanmi Lee, Danielle M. Maatouk, Brian D. Harfe

**Affiliations:** 1 Department of Molecular Genetics and Microbiology and the Genetics Institute, University of Florida, College of Medicine, Gainesville, Florida, United States of America; 2 Department of Cell Biology, Duke University Medical Center, Durham, North Carolina, United States of America; Texas A&M University, United States of America

## Abstract

Outgrowth and patterning of the vertebrate limb requires a functional apical ectodermal ridge (AER). The AER is a thickening of ectodermal tissue located at the distal end of the limb bud. Loss of this structure, either through genetic or physical manipulations results in truncation of the limb. A number of genes, including *Bmps*, are expressed in the AER. Previously, it was shown that removal of the BMP receptor *Bmpr1a* specifically from the AER resulted in complete loss of hindlimbs suggesting that Bmp signaling in the AER is required for limb outgrowth. In this report, we genetically removed the three known AER-expressed Bmp ligands, *Bmp2*, *Bmp4* and *Bmp7* from the AER of the limb bud using floxed conditional alleles and the *Msx2-cre* allele. Surprisingly, only defects in digit patterning and not limb outgrowth were observed. In triple mutants, the anterior and posterior AER was present but loss of the central region of the AER was observed. These data suggest that Bmp ligands expressed in the AER are not required for limb outgrowth but instead play an essential role in maintaining the AER and patterning vertebrate digits.

## Introduction

The vertebrate limb begins as a bud of lateral plate mesenchyme surrounded by surface ectoderm. In mice, over the course of ∼4 days this undifferentiated bud of tissue forms all structures found in a normal limb [1]. The molecular pathways responsible for the formation of the limb have been an active area of investigation for decades and a number of factors have been identified that are required for limb development.

Outgrowth of the limb is controlled by the Apical Ectodermal Ridge (AER). This structure resides at the distal end of the limb bud and is composed of a stratified columnar of ectodermal cells [2], [3]. Removal of this structure results in truncation of the forming limb [4], [5], [6]. A class of AER-specific factors required for limb bud outgrowth is *Fgfs* (*Fgf4*, *Fgf8*, *Fgf9* and *Fgf17*). *Fgf8* appears to be the major factor responsible for limb bud outgrowth since removal of this gene from the AER, but none of the other AER-expressed *Fgfs*, results in defects in limb patterning [7], [8]. Removal of both *Fgf4* and *Fgf8* results in an absence of limb bud outgrowth, indicating that *Fgf* genes can partially compensate for one another [9]. *Fgf8* is the most broadly and highly expressed *Fgf* gene in the AER, which may explain why removal of this gene, but none of the other AER-expressed *Fgfs*, produces a visible phenotype [10].

In addition to *Fgfs* a number of additional genes are expressed in the AER including members of the *Bmp* family. During limb formation, *Bmp* genes are expressed in the limb bud mesenchyme and AER. In the mesenchyme, they have been shown to be required for initiating chondrogenesis and limb patterning [11].

The early removal of all Bmp signaling in the AER, through conditional deletion of the Tgfβ receptor *Bmpr1a*, resulted in limb truncations [12]. These data indicate that this signaling pathway is required for limb outgrowth but the source of the BMP ligand could not be identified in these experiments. Previously, we reported that removal of *Bmp2* and *Bmp4* in the AER, surprisingly, did not cause limb truncations but instead resulted in polydactyly, an increase in cell proliferation, and a decrease in cell death in the limb suggesting that *Bmp* expression in the underlying mesenchyme or *Bmp7* expression in the AER may compensate for loss of *Bmp2/4* expression in the AER [13]. In these experiments *Bmp7* was still expressed in the AER and could potentially compensate for loss of *Bmp2* and *Bmp4* in this structure.

In the current study, triple mutant mice in which *Bmp2*, *Bmp4* and *Bmp7* were removed from the AER were created and analyzed. In all triple mutants, limb outgrowth occurred and a stylopod, zeugopod and autopod were present however; defects in autopod patterning were observed. These data suggest that *Bmps* expressed in the underlying mesenchyme can at least partially activate the Bmp signaling pathway in the AER and that AER-expressed *Bmps* are only required for digit patterning and not limb outgrowth.

**Figure 1:**
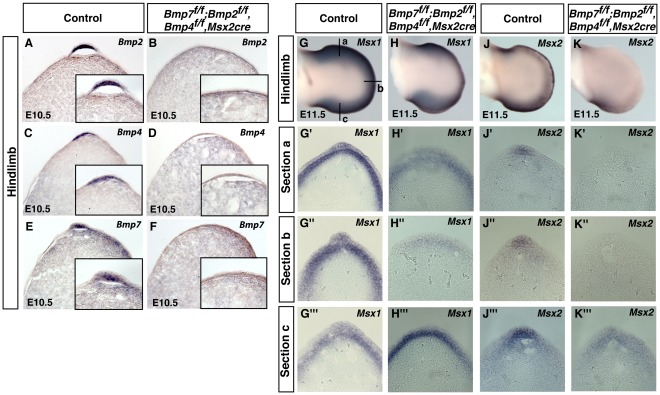
Removal of *Bmp2*, *Bmp4* and *Bmp7* from the same limb bud using the *Msx2-cre* allele. RNA *in situ* hybridization of *Bmp2, Bmp4* and *Bmp7* in wild type (A, C, E) and a triple mutant (B, D, F) hindlimb. *Bmp2, Bmp4* and *Bmp7* expression was removed in the triple mutants. Triple mutants (B, D, F) contained a thinner AER than wild type littermates. Whole mount and section RNA *in situ* hybridizations for *Msx1* and *Msx2* in E11.5 hindlimb buds (G-K). Anterior (section a), central (section b) and posterior (section c) sections of wild type (G and J) and the triple mutant (H and K) hindlimb buds are shown. In the anterior (G’-K’) and central (G”-K”) AER *Msx1* expression was downregulated and *Msx2* expression was absent. *Msx1* and *Msx2* expression persisted in the posterior AER (G”’-K”’). 20 µm transverse sections of E10.5 and E11.5 hindlimb buds are shown. Insets in A-F are close-up views of the respective AER.

## Materials and Methods

### Statement of Ethical Approval

This study was performed in strict accordance with the recommendations in the Guide for the Care and Use of Laboratory Animals of the National Institutes of Health. The protocol was approved by the Institutional Animal Care and Use Committee (IACUC) at the University of Florida (Protocol number: 201005047).

### Generation of Embryos that Lacked *Bmp2*, *Bmp4* and *Bmp7* in the AER

Animals were handled in accordance with the University of Florida Institutional Animal Care and Use Committee. Mice containing conditional floxed alleles of *Bmp2, Bmp4, Bmp7* or *Msx2-cre* have been described previously [11], [14], [15], [16], [17], [18]. *Bmp7^ f/+^*; *Bmp2^f/f^, Bmp4^ f/f^, Msx2-cre* and *Bmp7^ f/f^*; *Bmp2^f/f^, Bmp4^ f/f^, Msx2-cre* embryos were generated by mating *Bmp7^ f/+^*; *Bmp2^f/f^, Bmp4^ f/f^, Msx2-cre* males with *Bmp7^ f/f^*; *Bmp2^f/f^, Bmp4^ f/f^* females. Control embryos lacked the *Msx2-cre* allele and contained different combinations of floxed *Bmp* alleles. *Bmp2*, *Bmp4* and the *Msx2-cre* alleles are all located on the same chromosome. Recombination of the *Bmp* floxed alleles and *Msx2-cre* onto the same chromosome has been described previously [13]. Triple homozygous mice for all three floxed Bmp alleles (*Bmp7^ f/f^*; *Bmp2^f/f^, Bmp4^ f/f^*) did not contain any visible defects. All mouse strains were on a mixed genetic background.

### RNA *in situ* Hybridization, β-galactosidase Staining, and Skeleton Preparations

Whole-mount RNA *in situ* hybridization and β-galactosidase staining were performed as described previously [19], [20], [21]. Skeleton preparations were performed as previously described [22].

### Immunohistochemistry

Embryos were embedded in OCT and sectioned at 10 µm as described previously [23]. For immunohistochemistry, sections were stained with anti-CD44 (BD Biosciences) or with anti-?Np63 (Santa Cruz). A Cy3 conjugated secondary antibody (Jackson Immunoresearch Laboratories) was used. Images were taken using a Leica DMRE microscope (Leica Microsystems Inc.).

**Figure 2:**
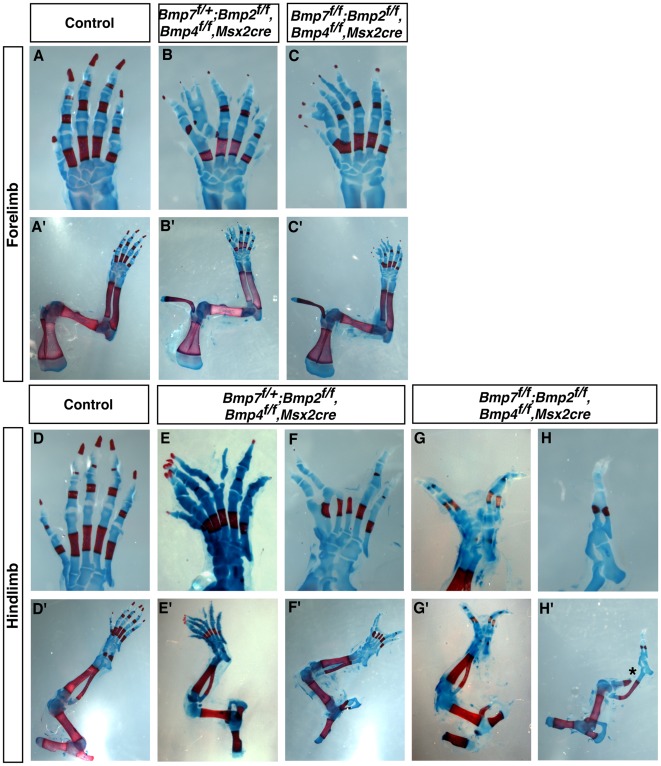
Removal of *Bmp2, Bmp4,* and *Bmp7* from the AER resulted in polydactyly, interdigital webbing, and split hand foot malformations. Skeleton preparation (A-H) of control and mutant fore- and hindlimbs of newborn mice (P0). Removal of either five of the six *Bmp* alleles from the forelimb or all known *Bmp* alleles from the forelimb AER (triple mutants) produced autopod patterning defects (A-C). Removal of five of the six *Bmp* alleles expressed in the AER (*Bmp7^ f/+^*; *Bmp2^f/f^, Bmp4^ f/f^, Msx2-cre*) resulted in the truncation of medial digits in hindlimbs (E and F). Removal of all six *Bmp* alleles from the AER (*Bmp7^ f/f^*; *Bmp2^f/f^, Bmp4^ f/f^, Msx2-cre*) produced severe truncation of medial and/or anterior digits (G and H). In the hindlimbs two examples are shown for each genotype. Truncation of the tibia was observed in 3/10 (30%) of triple mutant hindlimbs (“*” in H’). No other defects in proximal/distal patterning were observed in either the fore- or hindlimbs. A’-H’ are images of the entire limbs.

**Figure 3:**
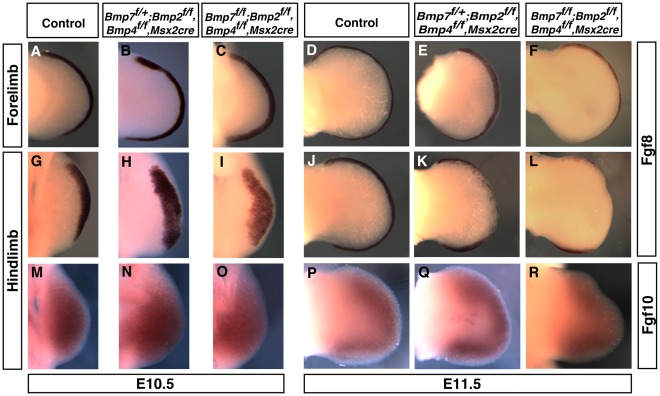
Removal of *Bmp2, Bmp4, and Bmp7* from the AER resulted in an initial broadening and then loss of *Fgf8* expression in the AER but normal *Fgf10* expression. In the forelimb, where the *Msx2-cre* allele instigates recombination after the AER has formed, a slight broadening of *Fgf8* expression was observed in the AER (A-C). In E10.5 hindlimb buds containing only a single allele of *Bmp7* (H; *Bmp7^ f/+^*; *Bmp2^f/f^, Bmp4^ f/f^, Msx2-cre*) or no functional *Bmp* alleles (I; *Bmp7^ f/f^*; *Bmp2^f/f^, Bmp4^ f/f^, Msx2-cre)*, *Fgf8* expression was broader throughout the AER. In E11.5 limb buds, *Fgf8* expression was severely decreased in the central and anterior AER in embryos that contained only a single allele of *Bmp7* (E and K; *Bmp7^ f/+^*; *Bmp2^f/f^, Bmp4^ f/f^, Msx2-cre*) or no functional *Bmp* alleles (F and L; *Bmp7^ f/f^*; *Bmp2^f/f^, Bmp4^ f/f^, Msx2-cre)*. In E10.5 hindlimb buds, *Fgf10* expression was not reduced in the limb bud mesenchyme that contained only a single allele of *Bmp7* or no functional *Bmp* alleles. The morphology of the distal limb bud was irregular in hindlimbs that contained only one functional *Bmp* allele (K, Q) or lacked all *Bmp* alleles (L, R). No differences in *Fgf10* expression were observed in the forelimbs (data not shown).

## Results

### Deletion of Floxed Alleles of *Bmp2, Bmp4* and *Bmp7* in the AER using the *Msx2-cre* Allele

To investigate the role(s) all three AER-expressed *Bmp* genes play in limb outgrowth and patterning, *Bmp2, Bmp4* and *Bmp7* were removed from the AER. Triple *Bmp2, Bmp4, Bmp7* AER knockout mice were created by crossing floxed animals to males carrying the *Msx2-cre* allele (see Materials and Methods). Throughout this report, “triple” knockouts refer to animals in which *Bmp2*, *Bmp4* and *Bmp7* were removed from the AER using the *Msx2-cre* allele. The *Msx2-cre* transgene is expressed in the ventral ectoderm and the AER and is expressed earlier in the hindlimb (24 somite stage) than in the forelimb (28 somite stage) [12], [14], [24]. All three Bmp floxed alleles produce null alleles upon CRE-inducible recombination of the floxed DNA sites [11], [25].

To determine if *Bmps* were removed from the AER in mutant animals, section RNA *in situ* hybridization on E10.5 embryos were performed using probes that were specific for the deleted region of either *Bmp2, Bmp4* or *Bmp7* (Figure 1). In mutant hindlimbs, where the *Msx2-cre* allele is active in the early limb bud ectoderm, *Bmp2*, *Bmp4* and *Bmp7* expression was abolished from the AER (Figure 1A–F). These data indicated that the *Msx2-Cre* allele successfully deleted all three *Bmp* genes in the same limb bud.

**Figure 4:**
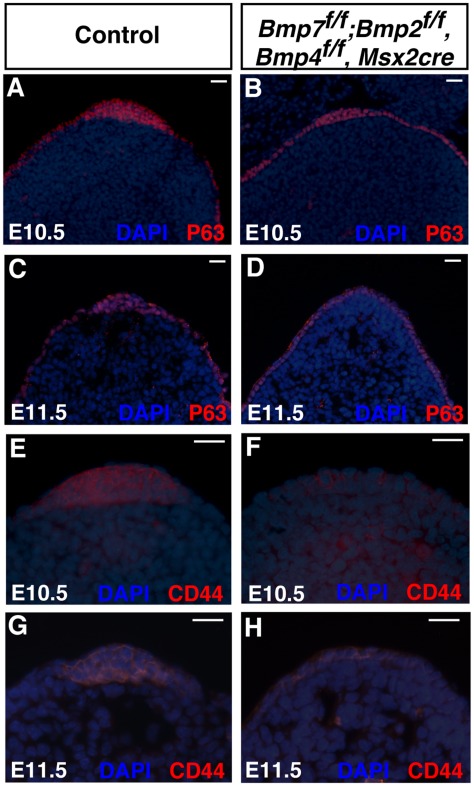
P63 and CD44 expression were decreased in triple mutants. (A-D) Immunostaining for ?Np63 on sections of wild type and *Bmp* triple mutants (*Bmp7^ f/f^*; *Bmp2^f/f^, Bmp4^ f/f^, Msx2-cre)*. ?Np63 was expressed in the AER and epithelial cells of E10.5 and E11.5 control (A and C) and triple mutant hindlimbs (B and D). In E10.5 triple mutants, the AER was elongated and thinner (B). By E11.5 (D), no visible AER was present. (E-H) Immunostaining for CD44 on sections of wild type (E and G) and triple mutant (F and H; *Bmp7^ f/f^*; *Bmp2^f/f^, Bmp4^ f/f^, Msx2-cre*) hindlimbs. CD44 expression was severely decreased in the *Bmp* triple mutants at E10.5 and E11.5. All sections shown are from the central AER. Scale bar = 20 µm.

### *Msx1* and *Msx2* Expression in the Central AER Requires *Bmp* Expression in the AER

BMP proteins are secreted signaling molecules and are expressed both in the limb bud mesenchyme and ectoderm during limb development. To determine if BMP signaling was altered in triple mutants, whole mount RNA *in situ* hybridization using probes against *Msx1* and *Msx2* was performed. Both of these transcription factors are known direct targets of the BMP signaling pathway [26]. In triple mutants, *Msx1* and *Msx2* expression was decreased in the anterior and central AER (Figure 1G–K). *Msx1* and *Msx2* expression remained in the posterior AER. In the mesenchyme, *Msx1* and *Msx2* expression were decreased in the central and anterior limb bud.

### *Bmp2, Bmp4* and *Bmp7* Expression in the AER is Required for Autopod Patterning but not Limb Outgrowth

Previously, we demonstrated that removal of *Bmp2* and *Bmp4* in the AER resulted in polydactyly, an increase in cell proliferation and a decrease in cell death in limb bud mesenchyme [13]. *Bmp7* was still expressed in the AER of these limb buds. In this report, we analyzed triple mutants in which *Bmp2*, *Bmp4* and *Bmp7* were removed from the same limb bud AER. In the forelimbs of embryos that contained AER null alleles for all six *Bmp* alleles (n = 10) or embryos in which only one functional *Bmp7* allele remained (n = 15), polydactyly, syndactyly and retention of interdigital tissue was observed in the autopods of all limbs examined (Figure 2 and Figure S1). No defects in limb outgrowth or in the stylopod or zeugopod were observed (Figure 2). In mutant hindlimbs that contained only one allele of *Bmp7*, ectrodactyly (n = 9/15) was observed (Figure 2E and F and Figure S1). In these limbs, the middle digits were truncated. In triple mutant hindlimbs, ectrodactyly (n = 7/10) and oligodactyly (n = 3/10) was observed (Figure 2G and H and Figure S1). Truncation of the anterior tibia was observed in 3/10 triple mutant hindlimbs (Figure 2H’).

### Removal of *Bmps* in the AER Resulted in an Initial Broadening of *Fgf8* Expression Followed by Loss of *Fgf8* Expression

To determine whether *Bmps* regulate the morphology of the AER, *Fgf8* expression was analyzed. *Fgf8* is expressed in the entire AER and is an excellent marker of AER morphology [27]. In embryos that contained only a single allele of *Bmp7* in the AER, *Fgf8* expression in the E10.5 hindlimb was broadened (Figure 3H). In triple mutant mice, AER broadening was seen in both the fore- and hindlimbs (Figure 3). By E11.5, a decease in *Fgf8* expression was observed in the central AER in hindlimbs of embryos that contained a single functional allele of *Bmp7* in the AER. In triple mutants that lacked all six *Bmp* alleles in the AER, *Fgf8* expression was absent throughout the central hindlimb AER and was decreased in both the anterior and posterior AER (Figure 3L). In the forelimb, a decrease but no gaps in *Fgf8* expression was observed throughout the AER of triple mutants (Figure 3F).

**Figure 5:**
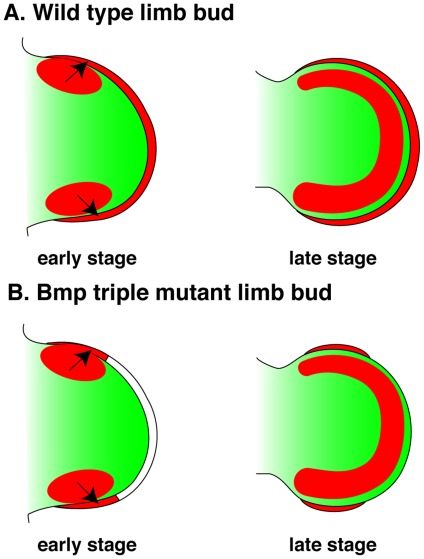
Proposed role for Bmp signaling in the AER. (A) In wild type limb buds, *Bmps* are initially expressed in the AER and the anterior and posterior mesenchyme. At later stages of development, *Bmp* expression is maintained in the AER and in the underlying mesenchyme. (B) Removal of *Bmp* ligands in the AER resulted in an abnormal expansion (early) and then loss (late) of the central AER. This resulted in defects in autopod patterning but not in limb outgrowth. During early limb bud development, BMP proteins produced in the anterior and posterior limb bud mesenchyme may partially rescue BMP signaling in these regions of the AER (arrows). The central AER appears to require early Bmp expression within this structure. During later development BMP proteins are expressed in the mesenchyme underneath the AER but expression at this time point in not sufficient to maintain a functional AER. Red  =  *Bmp* expression, green  =  limb bud mesenchyme, white  =  mutant AER.

### *Fgf10* Expression in the Mesenchyme does not Require *Bmp* Expression in the AER

*Fgf10* is expressed throughout the early limb bud [28], [29], [30]. During later limb development signaling from the AER is required to maintain *Fgf10* expression in the underlying limb bud mesenchyme. Mice null for *Fgf10* do not form limbs [31], [32]. Removal of the BMP receptor *Bmpr1a* in the AER resulted in initial expression of *Fgf10* throughout the limb bud mesenchyme during limb bud initiation but a subsequent loss of *Fgf10* during later development [12]. The inability to maintain *Fgf10* in *Bmpr1a* AER knockout mice was due to the lack of normal gene expression within the AER. To determine if expression of Bmp ligands within the AER were required for *Fgf10* expression, gene expression was analyzed in triple mutants. In triple mutants, *Fgf10* expression was initiated normally and expression was maintained during limb development (Figure 3M–R).

### *Bmps* Expressed in the AER are Required for Stratification of the AER

Analysis of *Fgf8* expression in triple mutants indicated that the central region of the AER may be absent by E11.5. To investigate the organization of the AER, two protein markers were examined. The first, p63 plays an important role in stratification of epithelial tissues [33], [34]. To determine if the phenotypes observed in *Bmp* mutant mice were caused by loss of p63, p63 protein expression was examined. A section through the AER of E10.5 and E11.5 triple mutant limb buds showed that removal of *Bmp* ligands in the AER did not affect p63 expression in the AER (Figure 4A–D). Analysis of p63 revealed that the AER was thinner and less stratified in triple mutants at E10.5 (Figure 4A, B). By E11.5 only a single p63-positive epithelial cell layer was observed that was indistinguishable from the surrounding epithelium (Figure 4C, D).

CD44 is a cell adhesion molecule and interacts with extracellular components [35]. Previous studies demonstrated that CD44 was highly expressed in the AER [36]. To determine whether expression of this cell adhesion molecule was altered upon removal of *Bmp* ligands in the AER, triple mutant embryos were examined using a CD44 antibody. A reduction of CD44 was observed in E10.5 and E11.5 embryos (Figure 4E–H). At E11.5, CD44 was expressed in a single layer of epithelial cell, consistent with observations made using the P63 antibody.

## Discussion

Early inactivation of the BMP receptor *Bmpr1a* in the hindlimb AER results in the absence of an AER and the inability to form a hindlimb [12], [37]. This result is in contrast to a previous report in which Noggin, a potent inhibitor of BMP signaling was used to block BMP signaling from the AER [38]. In these mice, limbs were formed but were characterized by syndactyly, postaxial polydactyly and dorsal/ventral patterning defects. It is possible that the differences in the observed defects were due to the timing of inactivation of the BMP signaling pathway; however in both cases the *Msx2* promoter was used to remove BMP activity. The phenotypes produced when *Bmp2*, *Bmp4* and *Bmp7* were removed from the same AER resembled *Msx2-noggin* limbs. Limb truncations were never observed (with the exception of loss of part of the tibia in 30% of triple mutant hindlimbs). Our experiments used the same *Msx2-cre* allele that was used to remove *Bmpr1a* suggesting that the concurrent removal of all known BMP ligands in the AER is not functional equivalent to removing the receptor that medicates BMP signaling in the AER.

Inactivation of the BMP receptor *Bmpr1a* in the forelimb resulted in broadening of the tips of distal phalanges [12]. The much less severe forelimb autopod defect is due to an early burst of *Bmpr1a* activity prior to CRE-mediated gene inactivation in the forelimbs [12]. Removal of *Bmp2*, *Bmp4* and *Bmp7* in the forelimb AER produced a more severe patterning defect than what was observed in the AER *Bmpr1a* receptor knockout. Our in situ expression data suggest that removal of AER-expressed *Bmps* resulted in a decrease in *Msx1/2* expression in the underlying mesenchyme in addition to decreased expression in the AER. It is possible that a decrease in BMP signaling in both the AER and mesenchyme upon inactivation of *Bmp2*, *Bmp4* and *Bmp7* in the forelimb AER causes autopod patterning defects that where more severe than what was observed upon removal of only the Bmp signaling pathway in the AER.

Removal of *Bmp* ligands from the AER were confirmed using RNA in situ probes that were specific for the deleted region of each *Bmp* allele. In addition, removal of BMP signaling was confirmed by analyzing expression of *Msx1* and *Msx2*, known targets of the BMP signaling pathway [26]. However, we cannot rule out the possibility that BMP activity remained in a few cells in the AER of triple mutant animals and that this potentially very low level of activity was sufficient for limb outgrowth. It is clear from our data that BMP expression in the AER is required for normal digit patterning and to maintain the structure of the central AER.

The BMPR1a receptor can bind additional TGFβ ligands besides BMP2, BMP4 and BMP7 [39]. In the limb bud AER a number of additional *Tgf*β genes are expressed besides *Bmps* [40], [41]. It is possible that complete loss of the hindlimb, which is observed upon removal of *Bmpr1a* from the AER [12], [37], is due to inactivation of the Bmp signaling pathway in addition to other TGFβ signaling pathways.

Individuals who contain a mutation in p63 have Split Hand Foot malformations (SHFM) [42]. Mice null for p63 had severe defects in epithelial stratification that affected AER formation, resulting in limb truncations [33], [34]. Removal of *Bmp2*, *Bmp4* and *Bmp7* from the limb bud AER resulted in defects in the autopod that resembled pattering defects found in SHFM. However, p63 was still expressed throughout the limb bud epithelium including the AER of E10.5 triple mutant embryos. These data suggest that the observed digit defects found in triple *Bmp* mutants are not caused by loss of P63.

In the central AER removal of *Bmp* genes resulted in a loss of *Fgf8* gene expression and, by E11.5, the AER. These data indicate that BMP expression within the AER is required not for AER formation but rather to maintain this structure during later limb development. Surprisingly, BMP signaling was not lost in the anterior and posterior AER, suggesting that during early stages of development BMP expression in the underlying mesoderm may be sufficient to activate the Bmp signaling pathway in these regions of the limb. All three *Bmp* genes are highly expressed in posterior limb bud mesenchyme at E10.5 with *Bmp7* also expressed in the anterior limb bud mesenchyme at this stage (Figure S2). Expression in these locations could potentially activate the Bmp signaling pathway in these regions of the limb (Figure 5). In the future, this hypothesis could be tested by concurrently removing *Bmp* genes from the AER using the *Msx2-cre* allele and from the posterior mesenchyme using the *Shhcre* allele [19]. Our data suggests that during normal limb development, *Bmps* expressed in the AER are required for digit patterning and BMP proteins produced in both the mesoderm and ectoderm of the limb bud can activate the Bmp signaling pathway in the AER.

## Supporting Information

Figure S1 **Removal of** ***Bmp2*** **,** ***Bmp4*** **,** ***Bmp7*** **results in retention of interdigital tissues.** Bright-field images of wild type (A and E) and mutant fore- and hindlimbs (B-D and F-H) of newborn mice. Interdigital webbing was observed in limbs containing only a single allele of *Bmp7* (B, F; *Bmp7^ f/+^*; *Bmp2^f/f^, Bmp4^ f/f^, Msx2-cre )* and in triple mutants that contained no *Bmp* alleles in the AER (C, D, G, H; *Bmp7^ f/f^*; *Bmp2^f/f^, Bmp4^ f/f^, Msx2-cre)*.
(TIF)

Figure S2 ***Bmp2, Bmp4*** **and** ***Bmp7*** **expression in wild type limb buds.** In E10.5 embryos (A-F), *Bmps* were expressed within the AER, directly underneath the AER and at elevated levels in the anterior and posterior limb bud mesenchyme (arrows). (G-L) By E11.5 Bmp ligands were expressed in the AER and throughout the limb bud mesenchyme.
(TIF)
