# Supplementary figures and images for: Bmp2, Bmp4 and Bmp7 Are Co-Required in the Mouse AER for Normal Digit Patterning but Not Limb Outgrowth
Source: PLoS One. 2012 May 25;7(5):e37826. doi: 10.1371/journal.pone.0037826 (PMC3360612; doi:10.1371/journal.pone.0037826)

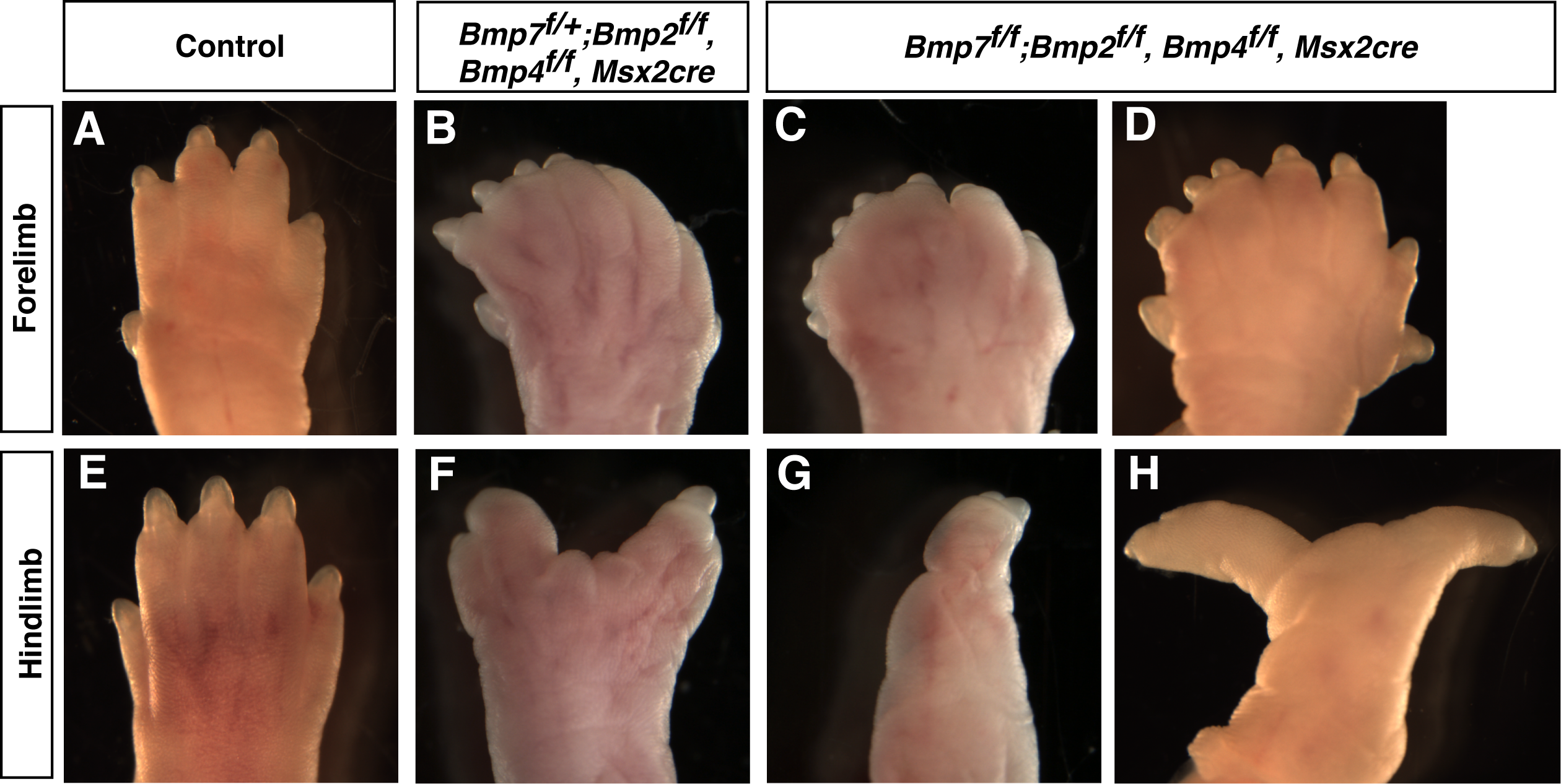

Supplement: Figure S1 — Removal of Bmp2 , Bmp4 , Bmp7 results in retention of interdigital tissues. Bright-field images of wild type (A and E) and mutant fore- and hindlimbs (B-D and F-H) of newborn mice. Interdigital webbing was observed in limbs containing only a single allele of Bmp7 (B, F; Bmp7 f/+; Bmp2f/f, Bmp4 f/f, Msx2-cre ) and in triple mutants that contained no Bmp alleles in the AER (C, D, G, H; Bmp7 f/f; Bmp2f/f, Bmp4 f/f, Msx2-cre). (TIF) [file pone.0037826.s001.tif]

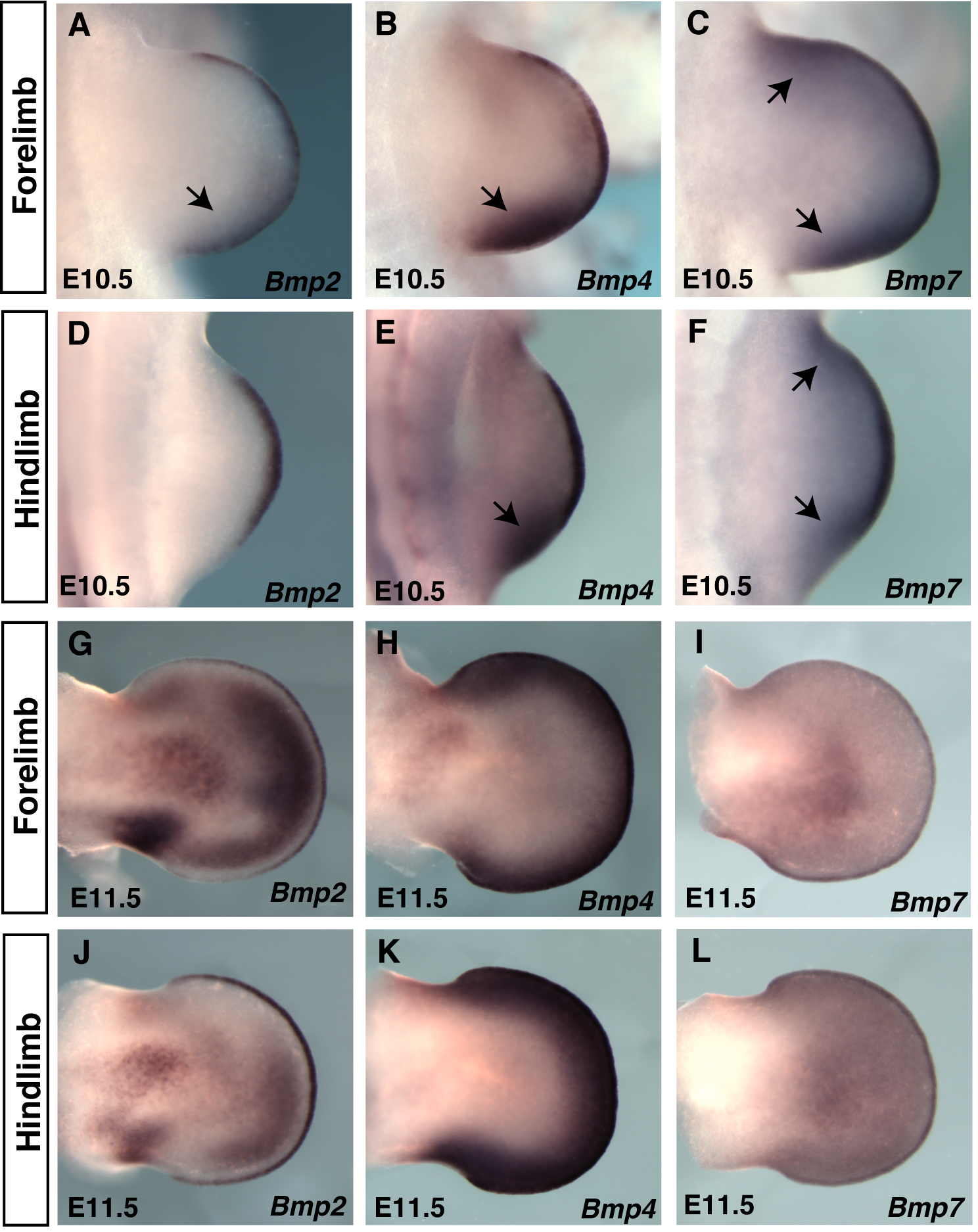

Supplement: Figure S2 — Bmp2, Bmp4 and Bmp7 expression in wild type limb buds. In E10.5 embryos (A-F), Bmps were expressed within the AER, directly underneath the AER and at elevated levels in the anterior and posterior limb bud mesenchyme (arrows). (G-L) By E11.5 Bmp ligands were expressed in the AER and throughout the limb bud mesenchyme. (TIF) [file pone.0037826.s002.tif]
